# The Properties of Linezolid, Rifampicin, and Vancomycin, as Well as the Mechanism of Action of Pentamidine, Determine Their Synergy against Gram-Negative Bacteria

**DOI:** 10.3390/ijms241813812

**Published:** 2023-09-07

**Authors:** Miran Tang, Deyi Zhao, Sichen Liu, Xiaotuan Zhang, Zhuocheng Yao, Hule Chen, Cui Zhou, Tieli Zhou, Chunquan Xu

**Affiliations:** 1 Department of Clinical Laboratory, Key Laboratory of Clinical Laboratory Diagnosis and Translational Research of Zhejiang Province, The First Affiliated Hospital of Wenzhou Medical University, Wenzhou 325035, China; tangmiran@163.com (M.T.); cicidurian@163.com (S.L.); zxt9289@163.com (X.Z.); yaozhuoc@163.com (Z.Y.); chlwakaka@163.com (H.C.); zhoucui0826@163.com (C.Z.); 2 Department of Medical Lab Science, School of Laboratory Medicine and Life Science, Wenzhou Medical University, Wenzhou 325015, China; zdy19980812@163.com

**Keywords:** gram-negative bacteria, pentamidine, linezolid, synergy, adjuvant, efflux pump, outer membrane, vancomycin

## Abstract

Combining pentamidine with Gram-positive-targeting antibiotics has been proven to be a promising strategy for treating infections from Gram-negative bacteria (GNB). However, which antibiotics pentamidine can and cannot synergize with and the reasons for the differences are unclear. This study aimed to identify the possible mechanisms for the differences in the synergy of pentamidine with rifampicin, linezolid, tetracycline, erythromycin, and vancomycin against GNB. Checkerboard assays were used to detect the synergy of pentamidine and the different antibiotics. To determine the mechanism of pentamidine, fluorescent labeling assays were used to measure membrane permeability, membrane potential, efflux pump activity, and reactive oxygen species (ROS); the LPS neutralization assay was used to evaluate the target site; and quantitative PCR was used to measure changes in efflux pump gene expression. Our results revealed that pentamidine strongly synergized with rifampicin, linezolid, and tetracycline and moderately synergized with erythromycin, but did not synergize with vancomycin against *E. coli*, *K. pneumoniae*, *E. cloacae*, and *A. baumannii*. Pentamidine increased the outer membrane permeability but did not demolish the outer and inner membranes, which exclusively permits the passage of hydrophobic, small-molecule antibiotics while hindering the entry of hydrophilic, large-molecule vancomycin. It dissipated the membrane proton motive force and inactivated the efflux pump, allowing the intracellular accumulation of antimicrobials that function as substrates of the efflux pump, such as linezolid. These processes resulted in metabolic perturbation and ROS production which ultimately was able to destroy the bacteria. These mechanisms of action of pentamidine on GNB indicate that it is prone to potentiating hydrophobic, small-molecule antibiotics, such as rifampicin, linezolid, and tetracycline, but not hydrophilic, large-molecule antibiotics like vancomycin against GNB. Collectively, our results highlight the importance of the physicochemical properties of antibiotics and the specific mechanisms of action of pentamidine for the synergy of pentamidine–antibiotic combinations. Pentamidine engages in various pathways in its interactions with GNB, but these mechanisms determine its specific synergistic effects with certain antibiotics against GNB. Pentamidine is a promising adjuvant, and we can optimize drug compatibility by considering its functional mechanisms.

## 1. Introduction

Multiple-drug or even pan-drug resistance and biofilm formation highlight the imminent threat that Gram-negative bacteria (GNB) represent. There is an urgent need to develop new therapeutic strategies to deal with these challenges. New, more sustainable treatment strategies for infections, drug repurposing, and drug combination strategies are receiving more attention [1]. Expanding the use of antimicrobials against Gram-positive bacteria (GPB) to treat GNB infections could be a promising strategy.

Pentamidine, synthesized in the late 1930s, is commonly used to treat trypanosomiasis, leishmaniasis, and fungal infections [2]. Recent studies have started to pay attention to its antibacterial activity [3,4,5]. In vitro drug susceptibility tests showed high minimum inhibitory concentrations (MICs) of pentamidine alone against GNB, indicating that its monotherapy is ineffective against GNB [4,5,6]. However, pentamidine has been proven to be effective in combination with antibacterial agents such as aminoglycosides, quinolones, carbapenems, and cephalosporins against GNB—even multidrug-resistant bacteria—including *Enterobacteriaceae*, *Acinetobacter baumannii*, and *Pseudomonas aeruginosa*. Interestingly, pentamidine can even be combined with narrow-spectrum antimicrobials that are only efficacious against GPB against GNB. The combination of pentamidine and erythromycin, rifampicin, and novobiocin showed synergistic activity against GNB [4]. The combination of pentamidine and rifampicin was reported to show potent in vitro activity against carbapenem- and/or colistin-resistant *Enterobacteriaceae* bacteria [6]. We also observed that combining pentamidine and linezolid had significant synergistic antibacterial and anti-biofilm activity, as well as in vivo efficacy in the clinical isolation of carbapenem-resistant enterobacteria (CRE) [7]. However, the properties of drugs that can be combined with pentamidine and the mechanisms of their synergistic antibacterial activity are still not completely understood.

The mechanisms of action of commonly-used antibiotics have mostly been clarified. For example, aminoglycosides and quinolones target intracellular proteins and nucleic acids, and carbapenems and cephalosporins inhibit the synthesis of the cell wall. That their MIC values for GNB were lower when combined with pentamidine leads us to hypothesize that pentamidine assists antibiotics in entering into GNB cells to exert their antibacterial effects. Of note, pentamidine was reported to be unable to synergize with colistin against *P. aeruginosa* [5]. Colistin can disrupt the outer membrane (OM) of GNB [8] and the absence of a notable synergistic effect with pentamidine leads us to hypothesize that pentamidine might similarly exert its effects specifically on the OM of GNB. Notably, certain Gram-positive-targeting antibiotics such as erythromycin, linezolid, and rifampicin inhibit bacterial protein synthesis by binding ribosomal subunits; vancomycin inhibits cell walls by targeting peptidoglycans [9]. Although these targets also exist in GNB [10], these drugs are almost ineffective against GNB. They are hindered from entering and achieving effective intracellular bactericidal concentrations by the highly impermeable OM of GNB with an abundance of lipopolysaccharide (LPS) and the efflux pumps [11,12]. However, under the synergistic effect of pentamidine, GNB is resensitized to these drugs, leading us to hypothesize that pentamidine acts specifically on the OM and efflux system of GNB, assisting antibiotics to enter into GNB cells to exert their antibacterial effects.

This study mainly elucidated the mechanism underlying the adjuvant activity of pentamidine to increase the susceptibility of GNB to rifampicin, linezolid, erythromycin, and tetracycline but not to vancomycin. Our investigation showed that pentamidine increased the OM permeability, but it did not demolish the outer and inner membranes, allowing specific antibiotics to enter; it dissipated the membrane proton motive force and inhibited the efflux pump, allowing the intracellular accumulation of antimicrobials that function as substrates of the efflux pump, such as linezolid. These processes finally resulted in metabolic perturbation and ROS production, killing the bacteria. These mechanisms of action of pentamidine on GNB indicate that it is prone to potentiating hydrophobic, small-molecule antibiotics such as rifampicin, linezolid, and tetracycline but not hydrophilic, large-molecule antibiotics such as vancomycin in this study.

## 2. Results and Discussion

### 2.1. Pentamidine Potentiates Rifampicin, Linezolid, Erythromycin, and Tetracycline except for Vancomycin against Multiple GNB

To determine which Gram-positive antibiotics can synergize with pentamidine against GNB, we used a panel of GPB antibiotics, including linezolid, rifampicin, erythromycin, tetracycline, and vancomycin to assess their combined antibacterial effects with pentamidine via checkerboard assays which indicate the synergistic or additive effect of drugs with a fractional inhibitory concentration index (FICI) of ≤1. Pentamidine strongly synergized with linezolid (FICI: <0.375, <0.375, <0.1875, 0.1875, 0.1563, 0.3125, 0.1563) and rifampicin (FICI: 0.375, <0.375, 0.75, 0.1875, 0.5), moderately synergized with erythromycin (FICI: 0.5, 0.5, 0.1563, <0.375) and tetracycline (FICI: <0.375, 0.5, 0.5, 0.5), but had no interaction with vancomycin (FICI: 2, 2, 2) against *E. coli*, *K. pneumoniae*, *E. cloacae*, and *A. baumannii* (Table 1). The different degrees and lack of synergistic effects suggested that diverse mechanisms are involved in the different combinations. Encouraged by these results, we next sought to clarify the specific mechanisms of the synergistic effects of pentamidine with certain antibiotics against GNB.

### 2.2. Pentamidine Preferentially Targets the Outer Membrane of GNB

**Figure 1:**
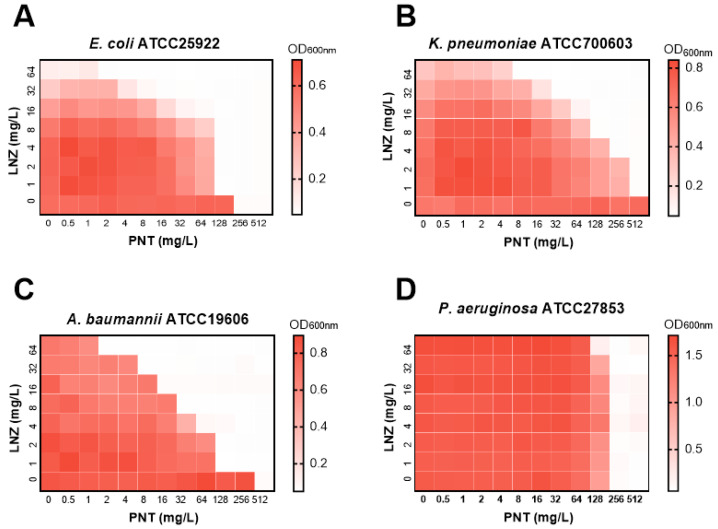
Heatmap of the checkerboards showing the combined effects of pentamidine with linezolid against representative ATCC Gram-negative strains of common clinical infectious species, including *E. coli* ATCC25922, FICI ≤ 0.375 (**A**), *K. pneumoniae* ATCC700603, FICI ≤ 0.375 (**B**), *A. baumannii* ATCC19606, FICI < 0.1875 (**C**), and *P. aeruginosa* ATCC27853, FICI = 2 (**D**). Dark regions represent higher cell density. PNT, pentamidine; LNZ, linezolid.

Previous work by our group had shown the potentiation of linezolid by pentamidine in CRE, including carbapenem-resistant *E. coli*, *K. pneumoniae*, and *E. cloacae* clinical isolates [7]. Xinxin Feng et al. also verified that pentamidine and linezolid have a highly synergistic effect against *E. coli* [14]. As such, in the following research on mechanisms, we mainly used linezolid as an example. To evaluate the spectrum of coverage by pentamidine in combination with linezolid against GNB, we examined their in vitro synergistic activity against representative ATCC GNB strains of common clinical infectious species, including *E. coli*, *K. pneumoniae*, *P. aeruginosa*, and *A. baumannii*. As shown in Figure 1, apart from *P. aeruginosa*, pentamidine synergized with linezolid in *E. coli*, *K. pneumoniae*, and *A. baumannii*, implying broad-spectrum synergistic activity.

**Figure 2:**
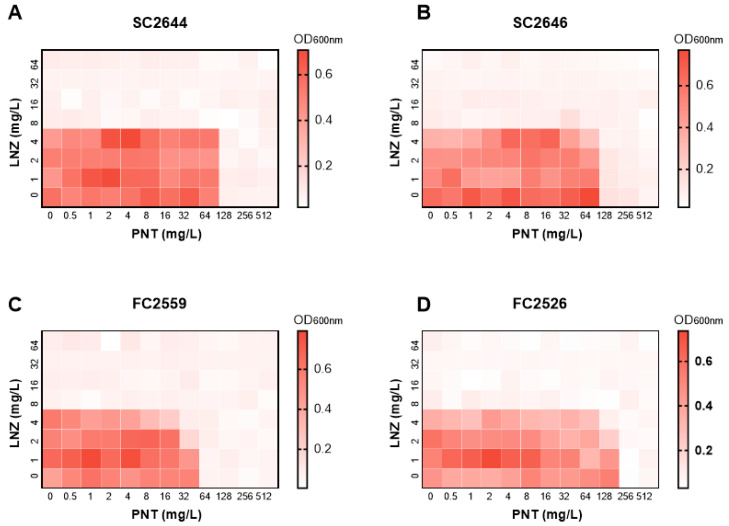
Chequerboard analysis showing the synergetic effect of pentamidine with linezolid in linezolid-resistant (LNZ^r^) Enterococcus strains (**A**) *Enterococcus faecium* SC2644; (**B**) *Enterococcus faecium* SC2646; (**C**) *Enterococcus faecalis* FC2559; (**D**) *Enterococcus faecalis* FC2526. No interaction is shown (FICI > 1). Dark regions represent higher cell density. PNT, pentamidine; LNZ, linezolid.

Linezolid exerts an antibacterial effect on GPB by binding the rRNA of ribosomal subunits to inhibit bacterial protein synthesis [15]. Mutations in 23S rRNA results in linezolid resistance (LNZ^r^), even if linezolid enters and accumulates in the bacterial cells. The checkerboard combination of pentamidine and linezolid on four clinically isolated LNZ^r^ Enterococcus strains (two *Enterococcus faecium* and two *Enterococcus faecalis*) showed they have no synergy on bacteria that are resistant to linezolid (Figure 2), highlighting that pentamidine did not exert antibacterial activity if linezolid does not have a bacterial target. Thus, we reasoned that linezolid is pivotal in bactericidal activity, and pentamidine functions as an adjuvant sensitizing GNB to it. This adjuvant effect cannot be seen in GPB without an OM so we speculated that the GNB OM is the target for pentamidine to exert its adjuvant effect.

### 2.3. Involvement of LPS Is Essential for Pentamidine to Disturb OM

**Figure 3:**
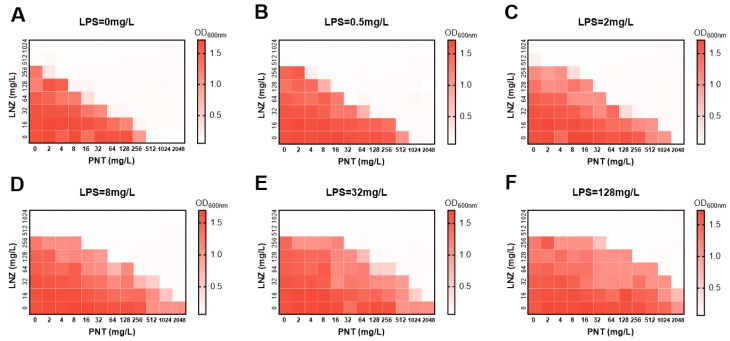
The effect of LPS on the synergetic effect of pentamidine with linezolid. The synergistic antibacterial effect of pentamidine/linezolid on clinically isolated carbapenem-resistant *K. pneumoniae* FK7921 with or without the addition of exogenous LPS (LPS = 0 (**A**), 0.5 (**B**), 2 (**C**), 8 (**D**), 32 (**E**), 128 mg/L (**F**)) detected by checkerboard assay. Dark regions represent higher cell density. PNT, pentamidine; LNZ, linezolid.

We proved that pentamidine preferentially targets the GNB OM; however, the specific location where pentamidine associates with the OM was not clear. The GNB OM contains heavily glycosylated lipids known as LPS. To investigate whether the participation of LPS is required for pentamidine to target and disturb the OM, we tested the effect of exogenous LPS on the combination effects of pentamidine/linezolid. Exogenous LPS decreased the synergistic antibacterial activities of pentamidine/linezolid against the carbapenem-resistant *K. pneumoniae* clinical isolate FK7921, compared with that without LPS (Figure 3). This finding indicates the involvement of LPS in the adjuvant activity of pentamidine.

**Figure 4:**
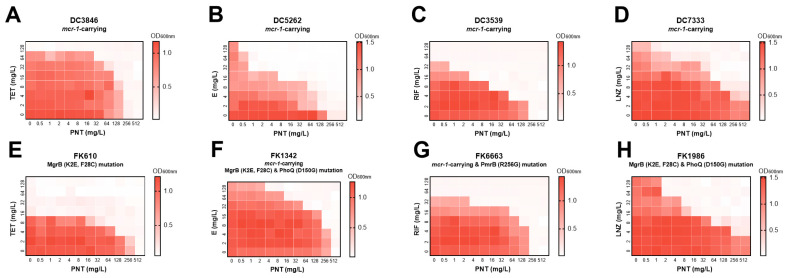
Heatmap of the checkerboards showing the combined effects of pentamidine with linezolid, rifampicin, erythromycin, and tetracycline against various clinical colistin-resistant Gram-negative strains. (**A**) Pentamidine/tetracycline on *mcr-1*-carrying *E. coli* DC3846, FICI = 0.5; (**B**) pentamidine/erythromycin on *mcr-1*-carrying *E. coli* DC5262, FICI = 0.1563; (**C**) pentamidine/rifampicin on *mcr-1*-carrying *E. coli* DC3539, FICI = 0.1875; (**D**) pentamidine/linezolid on mcr-1-carrying *E. coli* DC7333, FICI = 0.3125; (**E**) pentamidine/tetracycline on MgrB (K2E, F28C) mutation *K. pneumoniae* FK610, FICI = 0.5; (**F**) pentamidine/erythromycin on MgrB (K2E, F28C) and PhoQ (D150G) mutation as well as *mcr-1*-carrying *K. pneumoniae* FK1342, FICI <0.375; (**G**) pentamidine/rifampicin on PmrB (R256G) mutation and mcr-1-carrying *K. pneumoniae* FK6663, FICI = 0.5; (**H**) pentamidine/linezolid on MgrB (K2E, F28C) and PhoQ (D150G) mutation *K. pneumoniae* FK1986, FICI = 0.1563. Dark regions represent higher cell density. All showed synergy with FICI ≤ 0.5. PNT, pentamidine; LNZ, linezolid; RIF, rifampicin; E, erythromycin; TET, tetracycline.

Colistin resistance in GNB is most commonly attributed to modifications of LPS on the OM. These modifications are primarily mediated by mutations in the PmrA/PmrB and PhoP/PhoQ two-component regulatory systems, as well as the acquisition of the *mcr-1* gene. The *mcr-1* gene encodes the phosphoethanolamine transferase MCR-1, which modifies lipid A of LPS [16]. The synergy of pentamidine with linezolid, rifampicin, erythromycin, and tetracycline was maintained in colistin-resistant GNB with different molecular resistance mechanisms, such as the presence of *mcr-1* or *PmrA*/*PmrB* and *PhoP*/*PhoQ* gene mutations (Figure 4 and Table S1). Although not exhaustive, these data indicated that pentamidine might act with LPS regardless of its mutation or modification, which was consistent with a previously published study [4].

### 2.4. Pentamidine Increases the OM Permeability but Does Not Demolish the OM

**Figure 5:**
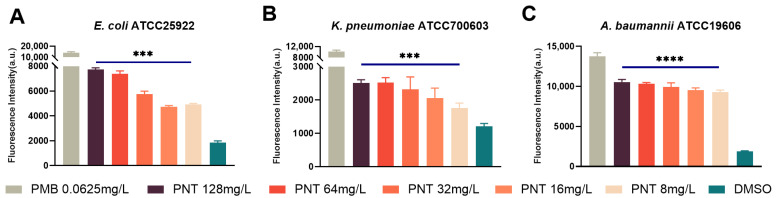
Outer membrane perturbation measured using N-phenylnaphthalen-1-amine (NPN) assay. *E. coli* ATCC25922 (**A**), *K. pneumoniae* ATCC700603 (**B**), or *A. baumannii* ATCC19606 (**C**) cells were pretreated with different concentrations of pentamidine (8–128 mg/L), with 0.0625 mg/L polymyxin B as positive control and DMSO as negative control, and then incubated with 30 μM of NPN. The NPN fluorescence intensity detected via a plate reader reflects the extent of OM perturbation. The significance between each pentamidine treatment group and the DMSO group was determined by using a two-sample *t*-test. ***, *p* < 0.001. ****, *p* < 0.0001. PNT, pentamidine; PMB, polymyxin B.

**Figure 6:**
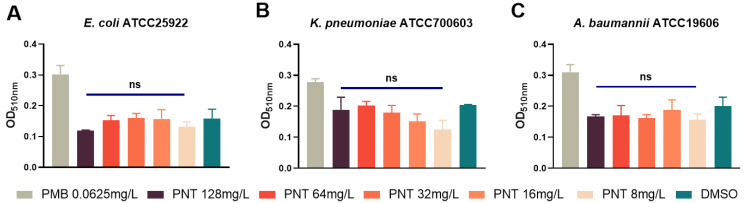
Outer membrane permeability determined by released periplasmic alkaline phosphatase (ALP). *E. coli* ATCC25922 (**A**), *K. pneumoniae* ATCC700603 (**B**), or *A. baumannii* ATCC19606 (**C**) cells were pretreated with different concentrations of pentamidine, with 0.0625 mg/L polymyxin B as positive control and DMSO as negative control; then, the enzyme release in the supernatant was determined by optical tests under 510 nm. The significance between each pentamidine treatment group and the DMSO group was determined by using a two-sample *t*-test. ns represents no significance.

The GNB OM was proven to be the target for pentamidine to exert its adjuvant effect. Therefore, we hypothesized that pentamidine may disturb the OM and thus enhance linezolid uptake. 1-*N*-phenylnaphthylamine (NPN) is a hydrophobic fluorescent probe that generates weak fluorescence signals in a hydrophilic environment but strong ones in a hydrophobic environment, such as the OM of bacteria [17]; thus, it is a useful probe to study cell membrane permeability. NPN is normally excluded from the intact bacterial membrane lipid bilayer by the OM barrier [18,19]. When the OM is damaged, NPN can penetrate and bind to lipids, resulting in a shift in its fluorescence excitation/emission spectra. As anticipated, pentamidine (8–128 mg/L) significantly elevated the NPN fluorescence in a dose-dependent manner (Figure 5), implying an OM permeabilization ability for pentamidine. Polymyxin B (PMB) vigorously damages the OM [20], and the sub-MIC of PMB can inhibit the OM [21]. Following the addition of the sub-MIC (0.0625 mg/L) of PMB, the MICs of rifampicin for *E. coli*, *K. pneumoniae*, *P. aeruginosa,* and *A. baumannii* were drastically reduced by about 32-fold (Table 2), implying that the intrinsic resistance to rifampicin of GNB is mainly due to the OM barrier effect. Pentamidine strongly synergized with rifampicin, with FICI values of 0.375, <0.375, and 0.375 against *E. coli*, *K. pneumoniae,* and *A. baumannii* (Table 1), respectively, implying its ability to disturb the OM. Interestingly, however, although the sub-MIC (0.0625 mg/L) of PMB also drastically reduced the MICs of vancomycin for *E. coli*, *K. pneumoniae*, *P. aeruginosa,* and *A. baumannii* by about 16-, 16-, 32-, and 8-fold, respectively (Table 2), pentamidine in combination with vancomycin showed no synergy (Table 1). This is consistent with the study by Brown and colleagues showing no synergy between pentamidine and vancomycin [4] and with the study by Martin and colleagues, which found that several bis-amidines with pronounced synergy with other antibiotics did not demonstrate any synergistic activity with vancomycin [22]. However, the reasons for these observations were not clarified in their studies. The OM lipid bilayer only allows hydrophobic small molecules to enter via passive diffusion [23,24]. The hydrophilicity of vancomycin hinders its ability to cross the lipid bilayer of the OM. Although nonspecific porins on the OM allow hydrophilic substances to enter the cell, there is a limit based on molecular size. Thus, vancomycin, as a large molecule [25], still encounters impediments. Only when the OM is highly disrupted or demolished can vancomycin freely reach its target, the cell wall, in the periplasm. PMB potentiated vancomycin against GNB due to its robust damage to the OM (Table 2), which is consistent with PMB having a synergistic effect with vancomycin [22]. The checkerboard assay of pentamidine and vancomycin showed that their combination had no synergy against GNB (Table 1). This indicated that pentamidine might not induce serious structural damage to the OM and therefore, the hydrophilic macromolecular vancomycin cannot be potentiated against GNB. This result was further ascertained by measuring the release of periplasmic proteins. Alkaline phosphatase is a macromolecular substance located in the periplasm of bacterial cells [26,27,28] and the quantity of alkaline phosphatase released reflects the extent of OM damage. No differences in the amount of alkaline phosphatase released between the pentamidine and negative control groups indicated that pentamidine-induced OM damage was insufficient to trigger indiscriminate alkaline phosphatase leakage (Figure 6). These data indicated that pentamidine might not induce substantial structural damage to the OM. The variations in synergy are attributed to the antibiotics’ capabilities to traverse the OM. Due to its large hydrophilic nature, vancomycin faces difficulty in traversing a slightly disrupted OM; in contrast, hydrophobic agents such as rifampicin, linezolid, erythromycin, and tetracycline [29] can effectively cross the mildly disturbed OM.

### 2.5. Pentamidine Does Not Disturb the Inner Membrane (IM)

**Figure 7:**
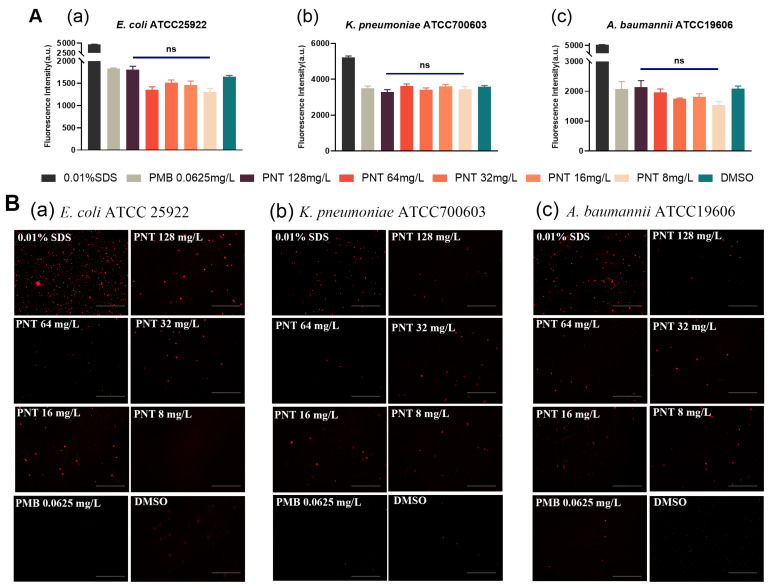
Inner membrane permeability determined by propidium iodide (PI) dye. *E. coli* ATCC25922 (**a**), *K. pneumoniae* ATCC700603 (**b**), or *A. baumannii* ATCC19606 (**c**) cells were pretreated with different concentrations of pentamidine (8–128 mg/L), with 0.01% *v*/*v* SDS as positive control, 0.0625 mg/L polymyxin B as outside negative control, and negative control only with DMSO, and then incubated with 50 mg/L PI. The PI fluorescence intensity was detected by a microplate reader (**A**). The significance between each pentamidine treatment group and the DMSO group was determined by using a two-sample *t*-test. ns represents no significance. The PI fluorescence intensity was also visualized by fluorescence microscopy (The scale bars represent 100 μm) (**B**). The PI fluorescence reflects the extent of IM perturbation.

**Figure 8:**
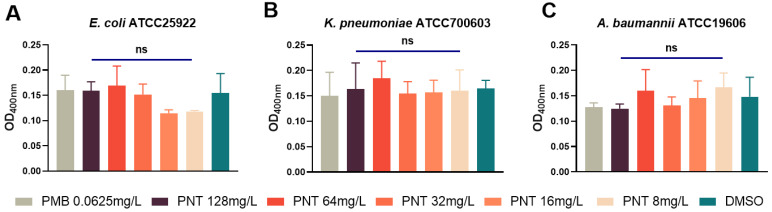
Inner membrane permeability determined by released cytoplasmic β-galactosidase. *E. coli* ATCC25922 (**A**), *K. pneumoniae* ATCC700603 (**B**), or *A. baumannii* ATCC19606 (**C**) cells were pretreated with different concentrations of pentamidine (8–128 mg/L), or 0.0625 mg/L polymyxin B as positive control and DMSO as negative control with DMSO, and then the enzyme release in the supernatant were determined by optical tests under 400 nm. The significance between each pentamidine treatment group and the DMSO group was determined by using a two-sample *t*-test. ns represents no significance.

GNB has two membranes. Many antibiotics, including linezolid and rifampicin, kill bacteria by crossing the IM and binding to bacterial intracellular targets [15]. Thus, we next explored whether pentamidine could disrupt the IM. Propidium iodide (PI), a cell-impermeable dye, cannot pass through an intact IM and can only bind to DNA and fluoresce when the membrane has been compromised [30]. Therefore, we investigated whether pentamidine allowed PI to breach the IM. The sub-MIC of PMB does not permeabilize the IM [20,21] so it was used as a negative control for IM penetration. Interestingly, exposure to pentamidine even at 128 mg/L did not increase the PI fluorescence, compared to the DMSO and PMB negative control groups (Figure 7A). Enhanced fluorescence imaging compared to the control group was also not observed (Figure 7B), indicating that pentamidine did not disrupt the IM. This can be supported by the result that β-galactosidase [29,31], an intracellular enzyme located in the cytoplasm of bacterial cells, was not released in higher quantities under pentamidine treatment compared to the PMB and DMSO control groups (Figure 8). From these data, we concluded that pentamidine does not disrupt the IM and enable antibiotics to cross it.

### 2.6. Pentamidine Dissipates the Membrane Proton Motive Force (PMF)

**Figure 9:**
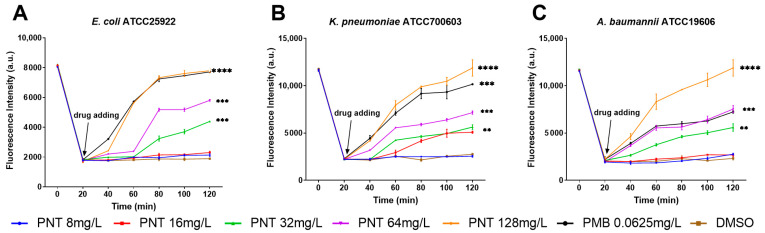
Membrane potential determined by fluorescence probe 3,3-dipropylthiadicarbocyanine iodide (DiSC3(5)) dye. *E. coli* ATCC25922 (**A**), *K. pneumoniae* ATCC700603 (**B**), or *A. baumannii* ATCC19606 (**C**) cells were preloaded with dye for 20 min, at which time the indicated concentration of pentamidine (8–128 mg/L) for experimental groups, the sub-MIC of PMB (0.0625 mg/L) for positive control, or no treatments (negative control) were added. The fluorescence intensity reflecting the membrane potential was detected every 20 min for a total of 120 min incubation. The significance between each pentamidine treatment group and the DMSO group was determined by using a two-sample *t*-test. **, *p* < 0.01; ***, *p* < 0.001; ****, *p* < 0.0001. PNT, pentamidine; PMB, polymyxin B.

One way to damage a membrane is to physically disrupt its barrier function [21]. Following confirmation that pentamidine did not destroy the IM physically, how linezolid can access cells seems to be particularly interesting. Membrane permeability is molecular size-selective and charge-selective [32]. Given that we have established that pentamidine did not structurally disrupt the molecular size selection of the membrane to elicit its adjuvant activity, our investigation shifted to addressing whether the synergy between pentamidine and linezolid was connected to pentamidine disrupting the charge selection of the membrane. The potential gradient (ΔΨ) is the main component of the transmembrane PMF [33]. Lipophilic membrane potential-sensitive fluorescent probe 3,3-dipropylthiadicarbocyanine iodide (DiSC3(5)) is taken up by the cells according to the magnitude of the ΔΨ [34]. When the cytoplasmic membrane is depolarized and disrupted, DiSC3(5) is released into the medium and the fluorescence increases. If a compound depolarizes the ΔΨ, the dye is released, increasing the fluorescence. We assessed the influence of pentamidine on the membrane potential using DiSC3(5). The permeabilizing antibiotic PMB was used as a positive control; its addition markedly increases DiSC3(5) fluorescence [35]. Similarly, pentamidine significantly increased the fluorescence of DiSC3(5) at concentrations of ≥32 mg/L, ≥16 mg/L, and ≥32 mg/L in *E. coli*, *K. pneumoniae,* and *A. baumannii*, respectively, compared with their DMSO-negative control groups (Figure 9), which indicated that pentamidine depolarized the ΔΨ and thus dissipated the membrane potential. Overall, our findings suggested that pentamidine does not affect the integrity but dissipates the PMF of the OM and IM.

### 2.7. Pentamidine Inhibits Efflux Pumps

**Figure 10:**
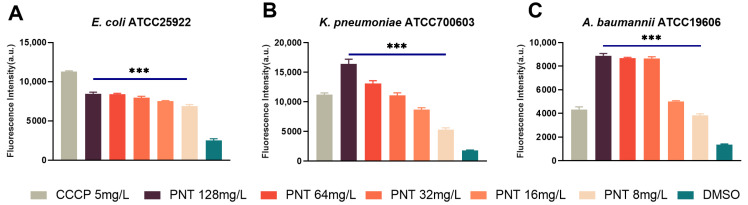
Efflux pump inhibition by pentamidine determined by acridine orange (AO) intracellular accumulation assay. *E. coli* ATCC25922 (**A**), *K. pneumoniae* ATCC700603 (**B**), or *A. baumannii* ATCC19606 (**C**) cells were pretreated with different concentrations of pentamidine, with 5 mg/L CCCP as positive control and DMSO as negative control and then the bacteria were dyed by AO. The fluorescence intensity reflects the intracellular AO accumulation and the efflux pump inhibition degree. The significance between each pentamidine treatment group and the DMSO group was determined by using a two-sample *t*-test. ***, *p* < 0.001. PNT, pentamidine; CCCP, carbonyl cyanide m-chlorophenylhydrazone.

**Figure 11:**
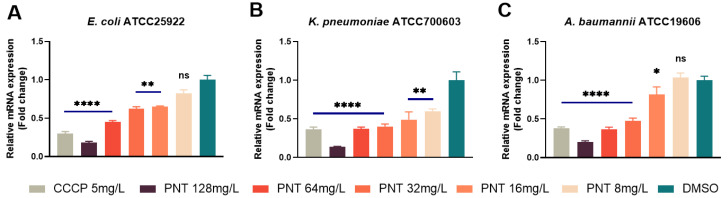
Efflux pump inhibition by pentamidine determined by efflux pump gene expression using qPCR. *E. coli* ATCC25922 (**A**), *K. pneumoniae* ATCC700603 (**B**), or *A. baumannii* ATCC19606 (**C**) cells were pretreated with different concentrations of pentamidine, with 5 mg/L CCCP as positive control and DMSO as negative control, and then the total RNA was extracted to detect the gene expression by qPCR. Negative control was standardized to 1. The significance between each pentamidine treatment group and the DMSO group was determined by using a two-sample *t*-test. ns, no significance; *, *p* < 0.05; **, *p* < 0.01; ****, *p* < 0.0001. PNT, pentamidine; CCCP, carbonyl cyanide m-chlorophenylhydrazone.

Carbonyl cyanide m-chlorophenylhydrazone (CCCP) is a well-known efflux pump inhibitor, especially for Resistance-Nodulation-Division (RND) efflux pumps. CCCP is a proton uncoupler known to collapse the membrane energy production and block the energy-dependent efflux pump [21,36]. The MICs of linezolid against the four GNB strains were significantly reduced by 32-, 2-, 8-, and 2-fold, respectively, when exposed to the sub-MIC (5 mg/L) of CCCP, whereas the presence of PMB showed negligible effects on the MICs (Table 2). These findings affirmed the substantial contribution of efflux to the innate resistance of GNB towards linezolid. Moreover, the enhanced OM permeability alone cannot entirely explain the remarkable synergistic effect observed between pentamidine and linezolid. Hence, we next investigated the potential inhibitory impact of pentamidine on efflux as a plausible mechanism underlying the potentiation of linezolid. Acridine orange (AO) intracellular accumulation was tested to assess the efflux inhibition [37]. AO is a substrate for efflux pumps, and efflux inhibition increases the intracellular accumulation of AO. CCCP, a well-known efflux pump inhibitor [38], was used as a positive control. As shown in Figure 10, the concentration-dependent promotion of intracellular AO accumulation by pentamidine was evident, mirroring a mode akin to that of CCCP. This result implied that pentamidine does indeed inhibit the efflux function of GNB. However, the precise mechanism by which this inhibition occurs remains elusive. On the one hand, the efflux pump fundamentally operates as a proton pump, sustained by the membrane PMF. The dissipation of the PMF leads to a depletion of the driving force essential for efflux pump activity, consequently triggering the deactivation of efflux pumps. Hence, it is plausible that the dissipation of pentamidine-induced PMF could account for the observed accumulation of AO or linezolid. On the other hand, the activities of efflux pumps are frequently subject to modulation by their substrate molecules [39]. Should pentamidine, functioning as an efflux pump substrate, engage in competition with AO or linezolid for binding to these pumps, the phenomenon described above becomes conceivable. Consequently, escalating doses of pentamidine, as demonstrated, would correspondingly elevate the intracellular accumulation of AO and linezolid. In both of these instances, it is conceivable that the expression of efflux pump genes does not undergo reduction and might even demonstrate an increase. RND is the main type of efflux pump in GNB [40,41]. The AcrAB–TolC of *E. coli* and *K. pneumoniae,* as well as AdeABC of *A. baumannii* are its major members [40,41]. As shown in Figure 11, pentamidine significantly inhibited the expressions of *AcrA* in *E. coli* and *K. pneumoniae*, as well as *adeB* in *A. baumannii*, respectively. Based on these findings, it becomes apparent that pentamidine functions as an efflux inhibitor, decreasing the activities and the gene expression of efflux pumps, thus resensitizing GNB to linezolid and rifampicin. Not only does the dissipation of the membrane potential deactivate efflux pumps, but the inhibition of efflux pump expression through some mechanism also reinforces the observation that pentamidine exhibited the most pronounced synergy with linezolid among the tested antibiotics.

The function of pumping harmful substances out of the cell is a major mechanism through which GNB resist lipophilic or amphiphilic antibiotics [42,43,44]. Its hydrophilicity indicates that vancomycin is not an efflux pump substrate. Notably, there was little change in the MICs of vancomycin in the presence of CCCP, indicating that vancomycin resistance was not related to efflux (Table 2). As such, efflux pump inhibition did not affect the susceptibility of GNB to vancomycin. All in all, pentamidine displays no synergy with vancomycin against GNB due to the macromolecular hydrophilicity of vancomycin.

### 2.8. Reactive Oxygen Species (ROS) Production Is the Endpoint for Bacterial Death under Pentamidine

**Figure 12:**
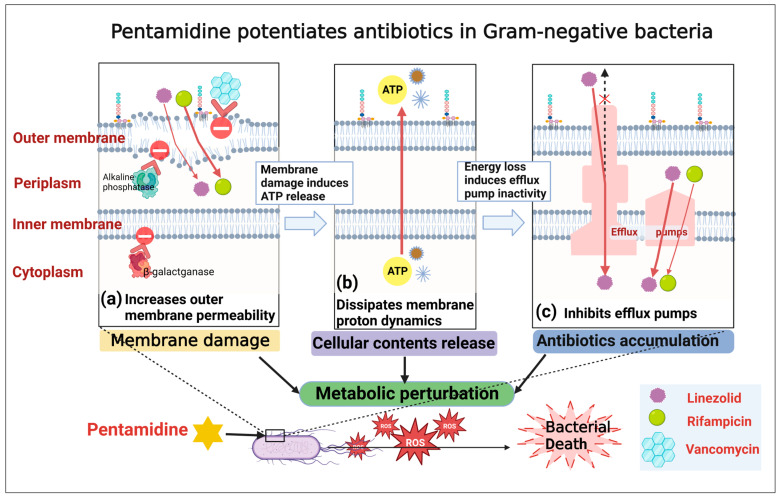
Schematic diagram depicting the processes of bacterial cell death under pentamidine in Gram-negative bacteria. (**a**) Pentamidine increases the outer membrane permeability; (**b**) dissipates the membrane potential, which results in leakage of cytoplasmic contents such as ATP and loss of cell energy that indirectly inactivates efflux pumps established dependently on transmembrane proton motive force (PMF); and (**c**) inhibits the efflux pumps. These processes perturb cellular metabolism and increase the intracellular accumulation of harmful substances including antibiotics such as linezolid and rifampicin, and thus promote the production of ROS which is the endpoint for bacterial death.

**Figure 13:**
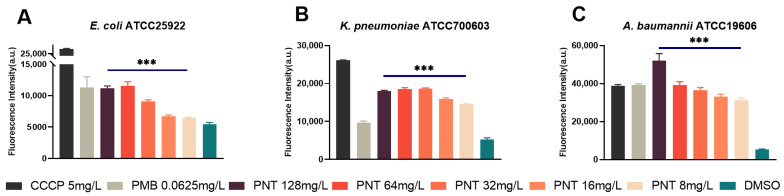
Intracellular ROS production by pentamidine determined using 7′-dichlorodihydrofluorescein diacetate (H2DCFDA). *E. coli* ATCC 25922 (**A**), *K. pneumoniae* ATCC700603 (**B**), or *A. baumannii* ATCC19606 (**C**) cells were pretreated with different concentrations of pentamidine, with 5 mg/L CCCP and 0.0625 mg/L polymyxin B as positive control and DMSO as negative control, and then the bacteria were dyed by H2DCFDA. The fluorescence intensity reflects the intracellular ROS accumulation. The significance between each pentamidine treatment group and the DMSO group was determined by using a two-sample *t*-test. ***, *p* < 0.001. PNT, pentamidine; PMB, polymyxin B; CCCP, carbonyl cyanide m-chlorophenylhydrazone.

The findings presented above illuminate the molecular underpinnings of pentamidine’s adjuvant function. Specifically, pentamidine was observed to enhance membrane permeability which results in leakage of cytoplasmic contents such as ATP and loss of cell energy that indirectly inactivates efflux pumps established dependently on transmembrane PMF; dissipate the PMF and concurrently inhibit efflux pumps which induces cellular contents release and antibiotics accumulation. These mechanisms collectively foster the intracellular accumulation of detrimental agents, including antibiotics. These processes perturb the cell’s metabolism, impair the respiration chain, and promote the production of ROS in bacterial cells, ultimately resulting in bacterial death (Figure 12) [45,46,47,48]. The dose-dependent elevation of ROS in the cell after pentamidine treatment (Figure 13) ascertained the hypothesis that ROS production is the endpoint for bacterial death under pentamidine.

## 3. Materials and Methods

### 3.1. Bacterial Strains, Agents and MICs

Clinical isolates were obtained from a tertiary teaching hospital (Wenzhou, China). Reference strains (*E. coli* ATCC25922, *K. pneumoniae* ATCC700603, and *A. baumannii* ATCC19606) were used in all mechanism assays. Antibiotics including rifampicin, linezolid, erythromycin, tetracycline, vancomycin, and polymyxin B (PMB) (Kangtai, Zhejiang, China) were used in this study. Pentamidine, carbonyl cyanide m-chlorophenylhydrazone (CCCP), and their solvent dimethyl sulfoxide (DMSO) were purchased from MedChemExpress (Junction, NJ, USA) and Sigma-Aldrich, USA (St. Louis, MO, USA). The broth microdilution (BMD) method was used to determine the MICs of all agents against all bacteria (Table S2). Sub-MICs of PMB (0.0625 mg/L) or SDS (0.01% *v/v*) (Solarbio, Beijing, China) were used as membrane permeators and CCCP (5 mg/L) as an efflux inhibitor [21]. Sub-MICs of CCCP (5 mg/L) and PMB (0.0625 mg/L) were added to serially two-fold-increased concentrations of linezolid, rifampicin, or vancomycin to determine their effects on the MICs of these antibiotics for multiple GNBs.

### 3.2. Checkerboard Assay

The synergy against multiple GNBs of pentamidine combined with antibiotics was examined by the checkerboard assay. The fractional inhibitory concentration index (FICI) was calculated for each antibiotic in each combination and interpreted for each combination against each test isolate as follows: FICI of ≤0.5, synergism, 0.5 < FICI < 1 additive effect, 1 ≤ FICI < 4 no interaction, and FICI ≥ 4, antagonism [13].

### 3.3. Membrane Permeability Assay

OM and IM permeability was measured by using the hydrophobic probe 1-N-phenylnaphthylamine (NPN, Aladdin, Shanghai, China) and propidium iodide (PI, Yuanye, Shanghai, China) uptake method, respectively, as earlier described with some modifications [30]. Equal volumes of bacterial solution (OD = 0.4–0.6) were treated with pentamidine (8–128 mg/L) for experimental groups, positive control with PMB (0.0625 mg/L) and SDS (0.01% *v/v*), respectively, or DMSO as negative control for 2 h. Bacteria were washed with GHEPES buffer (125 mM NaCl, 5 mM KCl, 1.3 mM MgSO_4_ 7H_2_O, 1.2 mM CaCl_2_ 2H2O, 20 mM HEPES, 5.8 mM D-glucose, pH 7.2–7.4) [49] and incubated with NPN (10 μM) or PI (50 mg/L) for 30 min. A multimode plate reader (VICTOR Nivo, PerkinElmer, Waltham, MA, USA) was used to measure the fluorescence at an excitation/emission wavelength of 350/420 and 535/615 nm, respectively. PI fluorescence was also visualized by fluorescence microscopy (Nikon, Tokyo, Japan).

### 3.4. Intracellular Enzyme Leakage Assays

Equal volumes of bacterial solution were treated with pentamidine (8–128 mg/L) for experimental groups, positive control groups with PMB (0.0625 mg/L) and SDS (0.01% *v/v*), respectively, and the negative control group without treatments, and incubated at 37 °C with shaking at 180 rpm for 24 h. Subsequently, the supernatant was collected by centrifuging, and the activities of alkaline phosphatase and β-1,4-galactosidase in the supernatant were determined with respective commercial kits (Solarbio, Beijing, China). Absorbances were monitored at 510 and 400 nm, respectively.

### 3.5. LPS Neutralization Assay

An LPS neutralization assay was administered to determine whether LPS is the necessary target of pentamidine [50]. Exogenous LPS at graded concentrations of 0, 0.5, 2, 8, 32, and 128 mg/L were added into the medium followed by checkerboard assays to detect the abilities of pentamidine to potentiate linezolid in *K. pneumoniae* isolate FK7921.

### 3.6. Membrane Potential Assay

The membrane potential was detected using a fluorescence probe 3,3-Dipropylthiadicarbocyanine iodide (DiSC3(5), Aladdin, China) as previously described with some modifications [21]. Cultures were washed with GHEPES buffer and then re-suspended in 30 μM DiSC3(5). The dye was allowed to be absorbed for 20 min, at which time the indicated concentration of pentamidine (8–128 mg/L), PMB (0.0625 mg/L) as positive control, and DMSO as negative control were added. The released fluorescence in the medium was detected every 20 min for a total of 120 min incubation at an excitation/emission of 622/670 nm.

### 3.7. Acridine Orange (AO) Intracellular Accumulation Assay

Overnight cultures were subcultured 1:100 in fresh LB (LB with 0.2% glucose) and grown to OD600 = 0.4–0.6. An aliquot of culture was treated with different concentrations of pentamidine (experimental groups), 5 mg/L CCCP (positive control), and DMSO (negative control) for 12 h. Cells were washed and subsequently resuspended with 30 μM of AO (MedChemExpress Co., Ltd., Monmouth Junction, NJ, USA). After incubation for 1 h, the fluorescence was tested an excitation/emission of 488/515 nm.

### 3.8. Quantitative Real-Time PCR (RT-qPCR) Analysis

To analyze the effect of pentamidine on the expression of efflux pump genes *acrA* (F 5′-GGCGATAAGTGGCTGGTGACAG-3′ and R 5′-CGCTTGCGGCTTGCTGGTTA-3′ for *E. coli* ATCC25922; F 5′-GGCGGAGTTCGACCAGATCAAC-3′ and R 5′-CGGCATAGTCGGTGGCGTTT-3′ for *K. pneumoniae* ATCC700603), and *adeB* (F 5′-GGCGGTACTTCCACCTGCAATT-3′ and R 5′-GTCACCTTGTGGCAACCCTTCA-3′ for *A. baumannii* ATCC19606), 50 μL of subculture was added to 5 mL of fresh LB broth supplemented with different concentrations of pentamidine (experimental groups), 5 mg/L CCCP (positive control) and DMSO (negative control). The tubes were incubated at 37 °C with shaking at 180 rpm for 24 h. Subsequently, the total RNA was extracted from the bacterial pellet cells by the Trizol method and the gene expression was determined using the SYBGreen (Takara, Dalian, China) method by the RT-qPCR detection system. The *16s RNA* gene was used as the internal control.

### 3.9. Reactive Oxygen Species (ROS) Detection

Bacterial intracellular ROS levels were detected using 2′,7′-dichlorodihydrofluorescein diacetate (H2DCFDA) (Solarbio, Beijing, China). The aliquot of bacterial culture was treated with pentamidine (8–128 mg/L) at 37 °C for 2 h. CCCP (5 mg/L) and PMB (0.0625 mg/L) were used as positive control, and the negative control was without treatment. Cells were pelleted and washed with GHEPES buffer and subsequently resuspended with 1 mL of 30 μM of H2DCFDA. After incubation for 1 h, 200 μL suspension was added to a black 96-well plate and the fluorescence was measured (excitation/emission 488/535 nm).

### 3.10. Statistical Analysis

Each experiment was performed in triplicate. The significance between each experimental group and the negative control group was determined by using a two-sample *t*-test or Mann-Whitney test. *p* < 0.05 (*), *p* < 0.01 (**), *p* < 0.001 (***), and *p* < 0.0001 (****) were statistically significant.

## 4. Conclusions

In conclusion, our findings indicate that pentamidine elevated OM permeability without disrupting the integrity of both the outer and inner membranes. This alteration exclusively permits the passage of hydrophobic small-molecule antibiotics, while hindering the entry of the hydrophilic large-molecule vancomycin. Furthermore, pentamidine dissipated the membrane’s PMF and suppressed efflux pump activity, thereby promoting the intracellular accumulation of antimicrobials that serve as efflux pump substrates, including linezolid. The precise mechanisms by which pentamidine interacts with GNB intricately govern its synergistic effects against GNB with specific antibiotics that share similar physicochemical attributes.

In this study, we highlighted the importance of the physicochemical properties of antibiotics and the specific mechanisms of action of pentamidine for the synergy of pentamidine–antibiotic combinations. Pentamidine engages with various pathways in its interactions with GNB, and these mechanisms underlie its specific synergistic outcomes with particular antibiotics against GNB. The combination effects of pentamidine–linezolid and pentamidine–vancomycin or other antibiotics diverge due to the variations in the molecular properties and modes of antibiotic action. Since pentamidine cannot demolish the membrane directly, it may be weaker than drugs such as polymyxins. However, if we judiciously analyze each drug’s characteristics and mode of action and strategically leverage complementary advantages, the synergistic potential of pentamidine as an adjuvant can be maximized. Additionally, the exploration of structural analogs, such as bis-amidines with similar yet reinforced structural configurations, holds promise for enhancing the synergistic efficacy of Gram-positive-specific antibiotics against Gram-negative pathogens [22,51].

## Figures and Tables

**Table 1:** FICI values for pentamidine in combination with Gram-positive antibacterials against Gram-negative bacteria.

Species	Strains	Monotherapy (mg/L)		Combination (mg/L)		FICI	Interpretation
		PNT	LNZ	PNT	LNZ		
*E. coli*	ATCC25922	256	>64	32	16	<0.375	Synergistic
*K. pneumoniae*	ATCC700603	>512	>64	128	8	<0.375	Synergistic
*A. baumannii*	ATCC19606	512	>64	32	8	<0.1875	Synergistic
*K. pneumoniae*	CR FK7921	512	512	64	32	0.1875	Synergistic
*E. cloacae*	CR CG1779	2048	1024	64	128	0.1563	Synergistic
*E. coli*	Col-R DC7333	>512	>128	32	32	0.3125	Synergistic
*K. pneumoniae*	Col-R FK1986	>512	>128	16	16	0.1563	Synergistic
*P. aeruginosa*	ATCC27853	256	>64	256	>64	2	No interaction
		**PNT**	**RIF**	**PNT**	**RIF**		
*E. coli*	ATCC25922	256	16	32	4	0.375	Synergistic
*K. pneumoniae*	ATCC700603	>512	32	64	8	<0.375	Synergistic
*A. baumannii*	ATCC19606	512	16	128	8	0.75	Additive
*E. coli*	Col-R DC3539	256	64	16	8	0.1875	Synergistic
*K. pneumoniae*	Col-R FK6663	256	64	64	16	0.5	Synergistic
		**PNT**	**E**	**PNT**	**E**		
*E. coli*	ATCC25922	256	128	64	32	0.5	Synergistic
*A. baumannii*	ATCC19606	512	128	128	32	0.5	Synergistic
*E. coli*	Col-R DC5262	256	>128	8	16	0.1563	Synergistic
*K. pneumoniae*	Col-R FK1342	512	>128	64	32	<0.375	Synergistic
		**PNT**	**TET**	**PNT**	**TET**		
*K. pneumoniae*	ATCC700603	>512	32	64	8	<0.375	Synergistic
*K. pneumoniae*	Col-R FK610	512	16	128	4	0.5	Synergistic
*E. coli*	Col-R DC3846	256	128	64	32	0.5	Synergistic
*K. pneumoniae*	Col-R FK6663	256	64	64	16	0.5	Synergistic
		**PNT**	**VA**	**PNT**	**VA**		
*E. coli*	ATCC25922	256	>128	256	>128	2	No interaction
*K. pneumoniae*	CR FK7921	512	128	512	128	2	No interaction
*A. baumannii*	ATCC19606	512	>128	512	>128	2	No interaction

NOTE: PNT, pentamidine; RIF, rifampicin; LNZ, linezolid; E, erythromycin; TET, tetracycline; VA, vancomycin. CR, Carbapenem-resistant; Col-R, Colistin-resistant; FICI, fractional inhibitory concentration index, FICI of ≤0.5, synergism, 0.5 < FICI < 1 additive effect, 1 ≤ FICI < 4 no interaction; and FICI ≥ 4, antagonism [13].

**Table 2:** Susceptibility (mg/L) of Gram-negative bacteria to rifampicin, linezolid and vancomycin.

Strain	RIF	RIF + PMB ^a^	RIF + CCCP ^b^	LNZ	LNZ + PMB ^a^	LNZ + CCCP ^b^	VA	VA + PMB ^a^	VA + CCCP ^b^
	RIF MIC (mg/L)			LNZ MIC (mg/L)			VA MIC (mg/L)		
*E. coli* ATCC25922	16	<0.5	8	1024	512	<0.5	1024	64	1024
*K. pneumoniae* ATCC700603	32	<0.5	8	512	256	<0.5	512	32	512
*P. aeruginosa* ATCC27853	16	<0.5	4	256	128	<0.5	512	16	512
*A. baumannii* ATCC19606	16	<0.5	8	1024	512	64	1024	128	1024

NOTE: ^a^ PMB = 0.0625 mg/L; ^b^ CCCP = 5 mg/L; RIF, rifampicin; LNZ, linezolid; VA, vancomycin. PMB, polymixin B; CCCP, carbonyl cyanide m-chlorophenylhydrazone.

## Data Availability

The datasets generated for this study are available on request to the corresponding author.

## Supplementary Materials

The supporting information can be downloaded at: https://www.mdpi.com/article/10.3390/ijms241813812/s1. Reference [52] is cited in the supplementary materials.
