# Supplementary material for: The Properties of Linezolid, Rifampicin, and Vancomycin, as Well as the Mechanism of Action of Pentamidine, Determine Their Synergy against Gram-Negative Bacteria
Source: Int J Mol Sci. 2023 Sep 7;24(18):13812. doi: 10.3390/ijms241813812 (PMC10530309; doi:10.3390/ijms241813812)
Supplement: Supplementary file 1 [file ijms-24-13812-s001.zip › ijms-2563578-supplementary.pdf]

Supplementary Material

Supplementary tables

Table S1 Molecular mechanisms of the used clinically isolated colistin-resistant bacteria.

**Strains**	**Isolates**	**MICs of colistin (mg/L)**	**Colistin-resistant mechanism**
E. coli	DC3846	8	*mcr-1*
	DC3539	16	*mcr-1*
	DC5262	≧64	*mcr-1*
	DC7333	4	*mcr-1*
K. pneumoniae	FK610	32	MgrB (K2E, F28C)
	FK6663	32	PmrB (R256G) + *mcr-1*
	FK1342	≧64	MgrB (K2E, F28C) + PhoQ (D150G) + *mcr-1*
	FK1986	16	MgrB (K2E, F28C) + PhoQ (D150G)

NOTE: The MIC value of colistin and colistin-resistant mechanism were determined in our previous work [1].

Reference

[1] Zhang, Y.; Lin, Y.; Zhang, X.; Chen, L.; Xu, C.; Liu, S.; Cao, J.; Zheng, X.; Jia, H.; Chen, L.; Zhou, T. Combining Colistin with Furanone C-30 Rescues Colistin Resistance of Gram-Negative Bacteria in Vitro and in Vivo. *Microbiol. Spectr.* **2021**, *9*, e0123121.

Table S2 MICs of tested agents against Gram-negative bacteria.

Tested agents		PMB	CCCP	PNT	LNZ	RIF	VA	E	TET
**Species**	**Isolates**	**MICs (mg/L)**							
E. coli	ATCC25922	0.25	10	256	>64	16	>128	128	16
	CR DC6581	0.25	10	256	>64	16	>128	128	16
	Col-R DC3846	8	10	256	>64	16	>128	128	128
	Col-R DC7333	4	10	>512	>128	32	128	>128	64
	Col-R DC3539	16	10	256	>128	64	128	>128	128
	Col-R DC5262	>64	10	256	>128	64	>128	>128	128
K. pneumoniae	ATCC700603	0.25	20	>512	>64	32	>128	128	32
	CR FK7921	0.25	20	512	512	32	>128	>128	64
	Col-R FK6663	32	20	256	512	64	>128	>128	64
	Col-R FK1986	16	20	>512	>128	32	128	>128	32
	Col-R FK1342	>64	20	512	>128	64	>128	>128	32
	Col-R FK610	32	20	512	>128	64	128	>128	64
E. cloacae	CR CG1779	0.25	20	2048	1024	32	>128	>128	64
P. aeruginosa	ATCC27853	0.25	10	256	>64	16	>128	128	16
A. baumannii	ATCC19606	0.25	20	512	1024	16	>128	128	64

Abbreviations: PMB, polymixin B; CCCP, carbonyl cyanide m-chlorophenylhydrazone; PNT, pentamidine; LNZ, linezolid; RIF, rifampicin; VA, vancomycin; E, erythromycin; TET, tetracycline. CR, Carbapenem-resistant; Col-R, Colistin-resistant.
